# The impact of parental psychological control on adolescents’ physical activity: the mediating role of self-control and the moderating role of psychological capital

**DOI:** 10.3389/fpsyg.2025.1501720

**Published:** 2025-05-14

**Authors:** Chao Song, Sha Ge, Weipeng Zhang

**Affiliations:** College of Sports Science, Tianjin Normal University, Tianjin, China

**Keywords:** parenting style, parental psychological control, physical activity of adolescents, self-control, psychological capacity, structural equation modeling

## Abstract

**Objective:**

Insufficient physical activity poses a significant threat to adolescent health. Parental psychological control, as a typical negative parenting style, exerts profound influences on health-related behaviors among adolescents. This study aims to identify and elucidate the specific mechanisms through which parental psychological control impacts adolescent physical activity. Furthermore, it explores the roles of self-control ability and psychological capital in mediating the relationship between parenting styles and adolescent physical activity. The findings of this research seek to provide guidance for family education practices.

**Methods:**

A survey was conducted on 1,010 students from the first year of junior high school to the second year of senior high school to assess parental psychological control, physical activity, self-control, and psychological capital. Data analysis and model validation were performed using SPSS 26 and AMOS 24.

**Results:**

A significant negative correlation was found between parental psychological control and adolescents’ physical activity levels. Self-control was observed to partially mediate this relationship, while psychological capital played a negative moderating role in the adverse impact of parental psychological control on adolescents’ physical activity.

**Conclusion:**

Parental psychological control had a negative impact on the level of physical activity among adolescents. Enhancing adolescents’ self-control and psychological capital could serve as potential strategies to mitigate this negative effect, encouraging adolescents to actively engage in physical activities. This paper provided a new theoretical perspective and practical basis for improving family education methods and promoting positive health-enhancing behaviors among adolescents.

## 1 Introduction

Physical activity, defined by the World Health Organization (WHO) as any bodily movement produced by skeletal muscles that results in energy expenditure beyond resting levels (World Health Organization, 2010), encompasses a wide range of behaviors, from structured exercise to everyday tasks like household chores. As a critical health-promoting behavior, physical activity plays a vital role in adolescents’ development, significantly contributing to their physical and psychological wellbeing (Lenze et al., 2024). However, WHO data highlights widespread physical inactivity among adolescents globally, posing a substantial threat to public health (Guthold et al., 2020). Physical inactivity is associated with a host of negative outcomes for both individuals and society, including increased risks for mental health issues such as anxiety and depression (Sluijs et al., 2021). Psychologically, insufficient motivation and low self-efficacy are key factors contributing to inactivity in this age group (Martins et al., 2021a). Identifying the barriers to physical activity, particularly those rooted in family dynamics, holds significant importance in promoting healthy behaviors among adolescents.

Family, as the cornerstone of adolescent development, exerts a profound influence on health-related behavioral choices through material support, emotional bonds, and value transmission (Rhodes et al., 2020). Theoretical frameworks such as Attachment Theory emphasize the role of the parent-child emotional connection in fostering autonomy, security, and self-efficacy—factors closely linked to the maintenance of health-promoting behaviors (Greenman and Johnson, 2022). In parallel, Bronfenbrenner’s Ecological Systems Theory underscores the dynamic interplay within the family microsystem as crucial to shaping adolescents’ health behaviors (Vaezghasemi et al., 2023). Among family-level factors, parenting strategies hold particular importance in influencing adolescents’ physical activity engagement. Positive parenting strategies, such as autonomy support, have been shown to enhance adolescents’ intrinsic motivation and foster healthy physical activity habits (Peng et al., 2024). Conversely, maladaptive parenting approaches, such as psychological control, can undermine adolescents’ motivation and participation in physical activity (Cozett and Roman, 2022). Psychological control, a parenting strategy characterized by behaviors such as inducing guilt or withdrawing love, has garnered considerable attention due to its negative implications for adolescent development (Barber et al., 1994). While behavioral control is often linked to positive outcomes such as better self-regulation, psychological control has been associated with emotional distress, reduced autonomy, and impaired self-regulation capacities (Grolnick and Pomerantz, 2009; Kakihara and Tilton-Weaver, 2009). These adverse effects may extend to health behaviors, as psychological control has been identified as a stress-inducing factor that contributes to a range of maladaptive outcomes, including psychological disorders, aggression, and behavioral dysfunctions (Yan et al., 2020). However, evidence remains insufficient regarding its specific impact on health-promoting behaviors such as physical activity.

Several studies within the domain of positive psychology have highlighted the positive contributions of supportive parenting behaviors to adolescents’ physical activity, particularly through the development of motivation and self-efficacy (Petersen et al., 2020; Dly et al., 2022; Yaffe and Levental, 2024). In contrast, restrictive or controlling parenting approaches have received comparatively less attention, leaving gaps in understanding how they influence adolescent health behaviors (Romm and Metzger, 2018; Romm et al., 2020). Physical activity, as a health-promoting behavior (Pascoe et al., 2020), requires individuals to possess strong autonomy and self-regulation (Englert and Rummel, 2016; Boat et al., 2024). Yet, these capacities may be impaired by parental psychological control (Pérez et al., 2021). Consequently, the extent to which psychological control diminishes physical activity engagement remains unclear.

This study aims to address these gaps by examining the relationship between parental psychological control and adolescents’ physical activity and exploring the mechanisms through which this influence operates. Specifically, it investigates the moderating and mediating roles of self-regulation and psychological capital. Psychological capital, a critical regulatory mechanism in coping with stress and adversity, has been shown to enhance health-promoting behaviors and physical activity levels (McDowell et al., 2019; Akdeniz Kudubes et al., 2022). Thus, it may serve as a buffer against the negative effects of parental psychological control. In summary, this research seeks to clarify the pathways linking parental psychological control to adolescents’ physical activity while advancing understanding of how family dynamics influence health-promoting behaviors. By uncovering key mechanisms such as self-regulation and psychological capital, the study aims to inform family-based interventions designed to foster a more active lifestyle among adolescents.

## 2 Theory and hypothesis

### 2.1 Parental psychological control and physical activity of adolescent

Physical activity refers to any bodily movement produced by skeletal muscles that results in energy expenditure exceeding resting levels. This encompasses various forms of exercise, recreational activities, occupational tasks, household chores, and modes of transportation (World Health Organization, 2010). It is a typical health-promoting behavior applicable to individuals of different ages and demographics. However, the level of physical activity among adolescents can be influenced by multiple factors, with the family environment, particularly parenting style, playing a significant role. To better understand how family environments and parenting styles interact with broader influences, Bronfenbrenner’s ecological systems theory (Veiga et al., 2023) provides a comprehensive framework. This theory highlights how adolescents’ physical activity is shaped by multiple environmental factors operating across five nested systems: the microsystem (e.g., family, peers), mesosystem (e.g., family-school interactions), exosystem (e.g., parental work conditions), macrosystem (e.g., cultural norms), and chronosystem (e.g., changes over time). Among these, the microsystem, which includes parenting styles such as psychological and behavioral control, plays a central role in shaping adolescents’ engagement in physical activity. Furthermore, the chronosystem emphasizes the importance of temporal factors, such as developmental changes during adolescence or evolving societal trends, which can influence both parenting practices and adolescents’ attitudes toward physical activity (Shah et al., 2023). Recognizing these dynamic interactions across systems is crucial for understanding cross-cultural similarities and differences in parenting.

Parental control comprises two primary types: behavioral control and psychological control (Inguglia et al., 2022). While behavioral control may yield some positive effects, psychological control—characterized by strategies such as guilt induction, love withdrawal, and authoritarian over control—is an intrusive and manipulative parenting approach (Barber, 1996). Unlike behavioral control, psychological control exerts influence by targeting adolescents’ emotions, values, and beliefs through coercive authority, and by employing tactics such as guilt induction and love withdrawal to enforce compliance with parental expectations and demands (Wang et al., 2007). This approach not only undermines adolescents’ intrinsic motivation and autonomy but also restricts their capacity for independent decision-making, subjecting them to substantial psychological stress and emotional challenges. As such, psychological control is widely recognized as a significant risk factor for the development of problem behaviors among children and adolescents (Barber et al., 1994).

The negative impact of parental psychological control on adolescents’ health-promoting behaviors was primarily reflected in two aspects: mental health and social skills. The weakening of these aspects might have further hindered adolescents’ participation in physical activities. Initially, psychological control greatly compromised adolescents’ mental health and emotional regulation (Yan et al., 2020), which in turn reduced their motivation to participate in physical activities and other health-promoting behaviors (Gu, 2022). This psychological control led to emotional disorders and externalizing issues in adolescents, causing social withdrawal (Lin et al., 2020). This kind of withdrawal behaviors reduced their motivation and opportunities for physical activities and restricted their ability to build social relationships through these activities, posing a barrier to developing overall health-promoting behaviors (Ike et al., 2020; Martins et al., 2021b; Qian et al., 2022).

Secondly, psychological control from parents, as a restrictive parenting strategy, severely constrained the development of adolescents’ self-efficacy (Wang et al., 2007) and autonomy (Loeb et al., 2021). Self-determination theory posited that fulfilling the three basic psychological needs of autonomy, competence, and relatedness could enhance self-motivation and engagement in various activities (Flannery, 2017). Due to reduced self-efficacy, adolescents might lack initiative and enthusiasm in adopting health-promoting behaviors such as proper diet and exercise. This change in psychological state could lead to a decrease in both the frequency and quality of their engagement in health-promoting behaviors like physical activities, thereby reducing the likelihood of adopting health-promoting behaviors (Zhang G. et al., 2024).

While psychological control has been extensively documented for its adverse effects on adolescents, it is important to recognize that not all forms of parental control are detrimental. Recent studies highlight that behavioral control, when implemented appropriately, may yield positive outcomes. For instance, parental monitoring has been associated with reduced risk behaviors and enhanced emotional regulation in adolescents (Commodari et al., 2024). Compared to psychological control, which undermines autonomy and self-motivation, behavioral control emphasizes structure and support, thereby fostering adolescents’ competence and engagement. Furthermore, research indicates that parenting practices and their outcomes may vary significantly across different cultural contexts, suggesting that cultural norms play a critical role in shaping the adaptability and effectiveness of parental control strategies (Novianti et al., 2023). These distinctions between psychological and behavioral control suggest that the evaluation of parental practices should consider both the specific type of control and the contextual factors influencing its effects.

**FIGURE 1:**
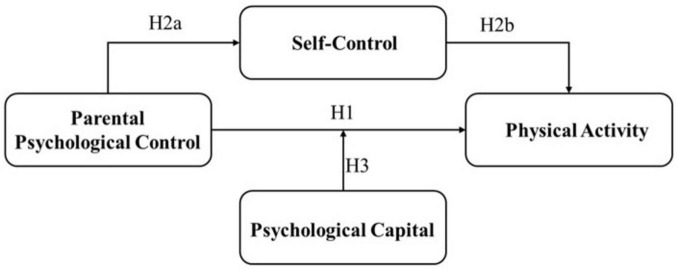
Conceptual study model.

In summary, psychological control exerted by parents indirectly affects adolescents’ willingness and ability to participate in physical activities by impacting their psychological health, emotional regulation capabilities, social behaviors, mental health, self-efficacy, and the development of autonomy. The combined effect of these factors may lead to a decrease in the frequency and quality of physical activity among adolescents, reducing the likelihood of adopting health-promoting behaviors. Based on the above, this study hypothesizes H1: Parental psychological control negatively impacts adolescents’ physical activity levels. (H1, Figure 1).

### 2.2 The role of self-control and psychological capital

Self-control, a crucial element of an individual’s personality system, referred to one’s capacity to regulate behavior, emotions, and cognitive processes in accordance with social norms or internal volition, encompassing both impulse control and self-discipline (Cai et al., 2021). Self-control is one of the most important indicators of adolescent socialization and a crucial factor in resisting internalizing issues such as stress and negative emotions, as well as externalizing problems like engaging in risky behaviors during adolescence.

Parental psychological control can restrict a child’s autonomy and emotional regulation skills, consequently impeding the cultivation of their self-control abilities. Attachment theory emphasized that the attachment relationship between children and their parents had a profound impact on their emotional and behavioral development. The quality of this attachment relationship directly affects the ability of children to regulate emotions and maintain psychological wellbeing. When the attachment relationship is threatened or insecure, it may lead to stress and negative emotions in children, consequently interfering with executive functions and impacting their capacity for self-control (Hofmann et al., 2012; Kerns et al., 2023). Parental control through emotional manipulation, including love withdrawal and guilt induction, disrupted children’s emotional security and self-regulation, and potentially triggered an accumulation of negative emotional stress (Pinquart, 2017). Furthermore, parental psychological control that deprived children of autonomy and decision-making abilities increased emotional stress and anxiety in children (Fang et al., 2024), ultimately having a negative impact on the development of self-control abilities in adolescents. Prolonged psychological control not only hindered the development of adolescents’ self-control abilities but also potentially weakened their ability to cope with challenges and reduced their motivation to engage in healthy activities (Pérez et al., 2021). This impact may have led adolescents to prefer a more sedentary lifestyle, such as excessive internet use, and decreased their participation in physical activities.

The impact of self-control on adolescents’ physical activity was reflected in two main aspects. Firstly, Adolescents’ motivation and persistence in exercise were influenced by self-control, which led to healthier behavioral choices being made, and consequently, higher levels of physical activity were promoted. Studies confirmed that self-control was particularly important for adolescents’ participation in high-intensity physical activities (Boat et al., 2024). It was found to have a significant impact on the selection and persistence of health-promoting behaviors (Hagger et al., 2019; Cobb-Clark et al., 2023), and was shown to positively predict levels of physical activity (Zhao et al., 2024). It was observed that adolescents with weaker self-control were more prone to sedentary behavioral patterns such as internet addiction (Xiang et al., 2019; Zhu et al., 2023), while individuals with stronger self-control demonstrated a greater ability to resist temptations and control impulses. These individuals were inclined to adopt healthier lifestyles, including physical activities (Moffitt et al., 2011), and were more likely to prioritize participation in sports activities over sedentary entertainment (Zhao et al., 2024). Secondly, self-control, as a psychological capability, was found to influence adolescents’ abilities to cope with stress and regulate emotions (Moffitt et al., 2011; Teixeira et al., 2012). Adolescents with stronger self-control were more effectively able to manage psychological stress resulting from inadequate parenting practices (Zhang S. et al., 2024), which was positively associated with their engagement in health-promoting behaviors, such as participating in physical activities (Boat et al., 2024). Consequently, adolescents’ self-control was identified as playing a crucial role in motivating and sustaining physical activity, guiding the selection of health-promoting behaviors, and maintaining health when confronted with adverse stressors.

Based on the above, parental psychological control was considered to weaken adolescents’ self-control capacity through increased emotional stress, autonomy deprivation, and negative emotions accumulation, thereby negatively impacting health-promoting behaviors such as physical activity. This study proposed the following hypothesis: adolescents’ self-control capacity mediated the relationship between parental psychological control and adolescents’ physical activity (H2, Figure 1). Specifically, parental psychological control was hypothesized to negatively affect adolescents’ self-control capacity (H2a), while enhanced self-control capacity in adolescents was expected to positively influence their level of physical activity (H2b). Adolescents’ psychological capital was found to play a crucial role in buffering against the detrimental impacts of parental psychological control, effectively mitigating its negative effects when they were confronted with such controlling behaviors (Inguglia et al., 2022). Psychological capital, a multidimensional construct comprising hope, self-efficacy, optimism, and resilience, represents an individual’s positive psychological state, with each component corresponding to confidence, self-capability recognition, a positive attitude, and the ability to withstand adversity and adapt, respectively (Maykrantz et al., 2021). This psychological resource was widely recognized as a critical regulatory mechanism in the face of stressors and crises, facilitating effective coping strategies (Maykrantz et al., 2021). Adolescents with higher levels of psychological capital were more likely to develop and maintain positive physical activity habits. Research confirmed that higher self-efficacy, optimism, a positive attitude, and greater resilience in the face of challenges all contributed to increased participation in physical activities among adolescents (Rasberry et al., 2011; Belcher et al., 2021). Moreover, elevated levels of psychological capital enabled children to maintain robust self-efficacy and resilience in the face of parental control, effectively buffering against its potential negative impacts (Carmona-Halty et al., 2022; Shengyao et al., 2024). Therefore, psychological capital exhibited a potential to be a mitigating factor against the adverse effects of parental psychological control. It could be served as a buffering mechanism, helping adolescents withstand the negative impact of such control. High levels of psychological capital were beneficial in helping adolescents maintain a positive attitude under the pressure of parental psychological control. The negative impact of such control on physical activity was mitigated, promoting their mental health and behavioral adaptation. We hypothesized that psychological capital would exert a negative moderating effect on the relationship between parental psychological control and adolescents’ physical activity (H3, Figure 1).

## 3 Materials and methods

### 3.1 Participation

The study was conducted among adolescents, with a deliberate focus on the congruence between the research content and the age-specific characteristics of the participants. To ensure a representative and diverse sample, stratified sampling was utilized, categorizing participants by grade level and gender. Questionnaires were administered across seven schools within the compulsory education sector, spread across six districts in Tianjin, a direct-controlled municipality in China. This methodological choice was intended to accurately reflect the diversity and nuances of the adolescent demographic, thereby bolstering the credibility and applicability of the study’s outcomes.

We distributed 1,350 paper questionnaires to students in grades seven through eleven, receiving 1,268 back. For accurate data entry, two teams independently numbered and entered the questionnaires under double-blind conditions, conducting cross-checks and quality control for completeness, consistency, homogeneity, and time anomalies. During the testing process, comprehensive guidance and training were provided to both investigators and participants to ensure accurate comprehension of the questionnaire content. The study employed anonymous responses to minimize social desirability bias and reduce potential overstatement of physical activity levels by participants. To enhance data authenticity and address recall bias, random samples underwent validation and supplementary interviews post-questionnaire, allowing cross-verification of self-reported activity levels against detailed verbal accounts. After screening, 1,010 valid questionnaires were included in the final analysis, with an effective response rate of 79.56%. Gender and age distribution details are in Table 1. Approximately 1,350 questionnaires were distributed to students across grades seven to eleven at the participating schools, with an allocation of 100-200 per grade level, yielding a total of 1,268 completed and returned responses. To ensure data entry accuracy, two research teams independently numbered and entered the questionnaires into the analysis system under double-blind conditions, performing cross-checks and quality control for completeness, logical consistency, homogeneity, and time anomalies. After screening, 1,010 valid questionnaires were included in the final analysis, yielding an effective response rate of 79.56%. The distribution of gender and age is detailed in Table 1. This study has been approved by the Ethics Committee of Tianjin Normal University (No: 2023122201). All guardians of the participating students have signed informed consent forms, and participation is entirely voluntary.

**TABLE 1:** Descriptive statistics of samples.

Variable	Option	Number (n)	Ratio (%)
Gender	Male	492	48.7
	Female	518	51.3
Grade (age)	7th grade	216	21.4
	8th grade	202	20.0
	9th grade	195	19.3
	10th grade	209	20.7
	11th grade	188	18.6
Family income (RMB/m outh)	Below 3,000	37	3.7
	3000-6,000	110	10.9
	6000-12,000	323	32.0
	12000-18,000	238	23.6
	18,000-24,000	154	15.2
	Above 24,000	148	14.7
Total		1,010	100.0

### 3.2 Variables

To investigate the impact and mechanisms of parental psychological control on physical activity levels of adolescent population, this study designates parental psychological control as the independent variable and adolescents’ physical activity levels as the dependent variable. Controlling for demographic factors such as parents’ occupation, education, family income, and adolescents’ age and gender, the study verifies the mediating effect of adolescents’ self-control and the moderating role of psychological capital in this relationship.

#### 3.2.1 Parental psychological control

Considering the pathways through which parental psychological control (PPC) operates, this study employed a specialized questionnaire developed by Wang et al. (2007) to measure this psychological phenomenon. The questionnaire consists of 18 items covering three dimensions: guilt induction (1-10), love withdrawal (11-15), and authority assertion (16-18). It uses a five-point Likert scale for scoring and reliable in this study (Cronbach’s Alpha of 0.89) and good construct validity (0.94). It has been widely used in related research, proving its effectiveness in practical applications (Tang et al., 2024). According to the scoring results, a higher score indicates a higher level of perceived parental psychological control experienced by the participants. The questionnaire uses a scale from 1 to 5 for each item, corresponding to the degree from “completely disagree” to “completely agree.”

#### 3.2.2 Adolescent physical activity level

In this study, the widely validated Physical Activity Rating Scale-3 (PARS-3) was used as the measurement tool (Liang, 1994; Duan et al., 2022; Zhang et al., 2023). This scale has been proven to exhibit excellent reliability and validity in assessing physical activity levels among adolescents (Cronbach’s α = 0.86, reliability = 0.82). The questionnaire required participants to recall their physical activity over the past month, including frequency, duration, and intensity, enabling a comprehensive evaluation of their physical activity levels. To minimize recall bias, the scale adopted a relatively short recall period (1 month), which not only captures overall activity levels but also ensures clarity in participants’ memories. Furthermore, the questionnaire was designed to be simple and easy to understand, reducing potential comprehension bias and improving the accuracy and consistency of responses. The PARS-3 encompasses three primary dimensions: duration, intensity, and frequency of exercise, with physical activity calculated as duration × intensity × frequency. Participants’ responses were rated on a five-point Likert scale, where the scores for exercise intensity and frequency ranged from 1 (light activity) to 5 (vigorous activity), and the scores for exercise duration ranged from 0 to 4.

#### 3.2.3 Self-control

Considering the compatibility of the measured indicators with physical activity levels, the study employed a questionnaire that was adapted from the Brief Self-Control Scale (BSCS) originally developed by Morean et al. (2014). The adaptation was authorized and conducted by Luo et al. (2021) to assess students’ self-control abilities (SC). The questionnaire comprised two dimensions: self-discipline (items 2, 4, 6, and 7) and impulse control (items 1, 3, and 5). The Cronbach’s α coefficients for the overall questionnaire and its two dimensions were 0.83, 0.85, and 0.86, respectively. The questionnaire utilized a 5-point Likert scale, ranging from “1” (not at all applicable) to “5” (completely applicable). Higher scores indicated stronger self-control abilities.

#### 3.2.4 Psychological capital

The study employed the Positive Psychological Capital Questionnaire (PPQ) developed by Kuo et al. (2010), which has been widely used to assess positive psychological capital in adolescents and adult college students (Li and Li, 2020), aiming to measure individuals’ psychological capital levels and their impact on mental health. The questionnaire, grounded in the concept of positive psychological capital as proposed by Luthans et al., utilized a 7-point Likert scale and comprised 26 items encompassing four core dimensions: self-efficacy, resilience, hope, and optimism. Higher scores on this scale reflected greater psychological capital and a more positive mindset. The PPQ had a Cronbach’s α of 0.90, demonstrating high internal consistency, with subscales scoring 0.86, 0.83, 0.80, and 0.76, further confirming its reliability. The Psychological Capital Scale demonstrated an acceptable model fit, indicating that its structural framework aligns well with the observed data (χ^2^/df = 2.450, GFI = 0.953, AGFI = 0.944, CFI = 0.973, TLI = 0.970, RMSEA = 0.038).

### 3.3 Statistical analysis

Data entry and organization were standardized and conducted using SPSS26, and confirmatory factor analysis was employed to examine the discriminant validity and common method bias of the measurement tools. The reliability of the questionnaire was assessed using Cronbach’s α, while validity was evaluated through the KMO sampling measure and Bartlett’s test of sphericity. Descriptive statistics and correlation analyses were also conducted. Demographic factors that might have influenced physical activity (such as child gender, grade level, and family monthly income) were included as control variables in regression analysis. AMOS24 software was used to test the research hypotheses and model through structural equation modeling, and mediation effects were examined using the Bootstrap method with 5,000 samples.

## 4 Results

### 4.1 Common method bias test and confirmatory factor analysis

To assess the presence of multicollinearity among the predictor variables, Variance Inflation Factor (VIF) values were calculated and confirmed to be within an acceptable range (VIF < 5), and tolerance values (1/VIF) were above the recommended threshold of 0.2. These findings collectively indicate no multicollinearity issues among the variables.

Both the Parental Psychological Control Scale and Self-Control Scale demonstrated acceptable model fit, indicating high consistency between the observed data and their respective theoretical frameworks. The fit indices for the Parental Psychological Control Scale (χ^2^/df = 4.281; GFI = 0.949; AGFI = 0.933; CFI = 0.966; TLI = 0.960; RMSEA = 0.057) and the Self-Control Scale (χ^2^/df = 4.623; GFI = 0.985; AGFI = 0.965; CFI = 0.987; TLI = 0.978; RMSEA = 0.060) further supported the structural validity of these measures. Before formulating the hypotheses, this study conducted a confirmatory factor analysis (CFA) to evaluate the fit of the measurement model. The results indicated a good model fit for the four factors (χ^2^/df = 3.959, GFI = 0.974, AGFI = 0.957, CFI = 0.963, TLI = 0.948, RMSEA = 0.054).

The analysis conducted through Harman single factor analysis to carry out the common method deviation analysis on all the valid data. As results shown, the first factor was 20.585%, smaller than the critical value of 40%, which meant that the common deviation method of the study was not remarkable. The results of the analysis are shown in Table 2. According to the results, CR > 0.7, AVE > 0.5 indicated that the degree of aggregation of the scale and the composite reliability was both acceptable. Furthermore, the AVE for several key constructs surpassed the inter-variable correlations within the matrix, signifying that the model demonstrated adequate discriminant validity.

**TABLE 2:** Results of correlation analysis, AVE, and CR.

Variables			Correlation analysis, AVE, and CR			
	**AVE**	**CR**	**1**	**2**	**3**	**4**
Parental psychological control	0.647	0.840	1			
Self-control	0.632	0.774	–0.302**	1		
Psychological capital	0.545	0.826	–0.006	0.026	1	
Physical activity			–0.423**	0.358**	–0.041	1
AVE mean root value			0.804	0.795	0.738	

**p < 0.01.

The correlation coefficients of these variables are presented in Table 2. Parental psychological control was found to have significant negative correlations with both the self-control scale and physical activity (*P* < 0.01), with correlation coefficients of –0.302 and –0.423, respectively, in the results of the correlation study, which presented an inverse relationship between the intensity of parental psychological control and both self-control and physical activity level of adolescent. A positive association was found between self-control and the physical activity level of adolescents (*P* < 0.01, *r* = 0.358), indicating that, all else being equal, higher levels of self-control were significantly associated with increased physical activity. Psychological capital presented no significant correlation with parental psychological control and physical activity (*P* > 0.05).

### 4.2 Descriptive statistics

Descriptive statistics of this study were presented in Table 1. A total of 1,010 survey participants were included, comprising 492 males (48.7%) and 518 females (51.3%). Participants aged 12-19 were distributed by grade: 216 in 7 h grade (21.4%), 202 in 8th grade (20.0%), 195 in 9th grade (19.3%), 209 in 10th grade (20.7%), and 188 in 11th grade (18.6%). Due to preparations for college entrance exams, 12th-grade students were not surveyed. Monthly household income distribution was as follows: below 3,000 yuan (3.7%), 3,000-6,000 yuan (10.9%), 6,000-12,000 yuan (32%), 12,000-18,000 yuan (23.6%), 18,000-24,000 yuan (15.2%), and above 24,000 yuan (14.7%). Overall, the demographic distribution of the sample was reasonable and representative.

Table 3 presented the descriptive statistics for the key observed variables in this study, including parental psychological control, self-control, psychological capital, and physical activity. The results were shown as follows: the total score for parental psychological control was found to have had a mean of 45.25 and a standard deviation of 24.75; the total score for self-control was found to have had a mean of 9.00 and a standard deviation of 3.00; the total score for psychological capital was found to have had a mean of 36.00 and a standard deviation of 12.00; the total score for physical activity was found to have had a mean of 30.00 and a standard deviation of 15.00. It should be noted that 69.10% of the adolescents surveyed were participants in moderate or higher levels of physical activity, which represented an increase compared to previously reported levels (Guthold et al., 2020).

**TABLE 3:** Descriptive statistics of variables.

Variable		Number, (N)	Min	Mix	Mean	SD
Parental psychology control	Total	1,010	18.00	90.00	45.25	17.23
	Guilt induction	1,010	10.00	50.00	25.24	10.81
	Love withdrawal	1,010	5.00	25.00	11.36	5.364
	Authority assertion	1,010	3.00	15.00	8.64	3.575
Self-control	Total	1,010	7.00	35.00	23.42	5.93
	Self-discipline	1,010	3.00	15.00	9.27	3.07
	Impulse control	1,010	4.00	20.00	14.16	3.70
Psychological capital	Total	1,010	22.00	154.00	95.85	29.10
	Self-efficacy	1,010	3.00	91.00	57.28	17.36
	Resilience	1,010	7.00	49.00	29.088	10.30
	Hope	1,010	6.00	42.00	28.76	8.76
	Optimism	1,010	6.00	42.00	28.19	9.19
Physical activity	Total	1,010	1.00	125.00	40.31	30.82
	Low level	312	1.00	18.00	10.04	5.61
	Median level	320	20.00	40.00	30.13	6.52
	Vigorous level	378	45.00	125.00	73.90	22.42

### 4.3 Hypothesis testing

#### 4.3.1 Assessment of model fit in mediation effect analysis

In this study, four key variables were designed: parental psychological control (3 dimensions, 18 items), self-control (2 dimensions, 7 items), psychological capital (4 dimensions, 26 items), and physical activity. All variables were assessed through self-reported questionnaires. Based on the research hypotheses, the model was employed to examine the mechanism by which parental psychological control influenced adolescent physical activity. The structural model was generated. Results showed an excellent fit to the data (χ^2^/df = 4.968, GFI = 0.989, AGFI = 0.967, CFI = 0.984, TLI = 0.967, RMSEA = 0.063).

**FIGURE 2:**
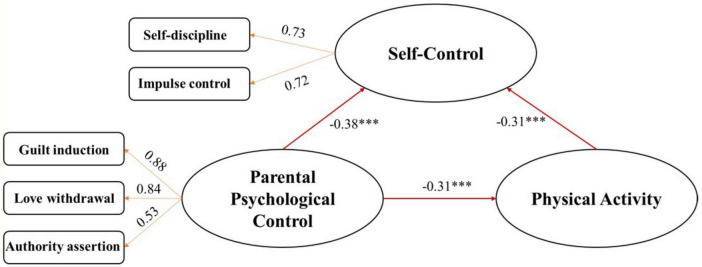
Parental psychological control—self-control—physical activity mediation model.

As results shown in Table 4 and Figure 2, parental psychological control significantly and negatively predicted self-control (*P* < 0.05, β = –0.282 < 0), indicating that decreased psychological control was associated with enhanced self-control among adolescents. Self-control positively predicted physical activity (*P* < 0.05, β = 1.874 > 0), suggesting that adolescents with greater self-control were more likely to engage in physical activity. Furthermore, psychological control negatively predicted physical activity (*P* < 0.05, β = –10.624 < 0), implying that lower levels of psychological control were linked to increased physical activity levels, with all other variables held constant.

**TABLE 4:** Test of regression coefficient of each path of the modified model.

Path	Unstd.estimate	Std.estimate	S.E.	C.R.	*P*
Self-control ← parental psychological control	–0.282	–0.379	0.032	–8.726	***
Physical activity ← self-control	14.335	0.312	1.874	7.65	***
Physical activity ← parental psychological control	–10.624	–0.311	1.187	–8.949	***

***p < 0.001.

Bootstrap method was employed to test the mediating effect of the model, with 5,000 resampling iterations and 95% confidence intervals. The results (see Table 5) indicated that the indirect effect was –0.118, with a 95% confidence interval ranging from –0.165 to –0.080, not encompassing zero, thereby demonstrating a statistically significant mediating effect. According to Cohen’s (Cohen, 2013) effect size criteria, the indirect effect value of –0.118 suggests a weak to moderate strength, indicating that the mediating variable plays a limited but non-negligible role in the causal pathway. The direct effect was –0.311, with a 95% confidence interval from –0.384 to –0.234, also not including zero, which denoted a significant direct effect. The absolute value of the direct effect (–0.311) approaches a moderate strength, indicating that the independent variable has a relatively substantial influence on the dependent variable.

**TABLE 5:** Result of bootstrap mediation effect testing.

Path	Effect value	Std. estimate	Bootstrapped 95% BC CI	
			**Lower**	**Upper**
Parental psychological control—self-control	–0.118	0.022	–0.167	–0.081
Self-control—physical activity				
Parental psychological control—physical activity	–0.311	0.038	–0.384	–0.234
Parental psychological control—self-control—physical activity	–0.429	0.032	0.032	–0.364

#### 4.3.2 Moderation effect analysis

In the regression analysis, grade, gender, and monthly household income were included as control variables to explore the moderating role of psychological capital on the negative impact of parental psychological control on adolescents’ physical activity levels (Table 6). The model, with an R-squared of 0.238, accounted for 23.8% of the variance in activity levels. An *F*-value of 52.271 confirmed the model’s significance at the 0.001 level. Results indicated that parental psychological control exerted a significant negative effect on physical activity (β = –0.374, *P* < 0.05), while no significant effect was found for psychological capital itself (*P* > 0.05). Moreover, a significant interaction effect between parental psychological control and psychological capital was identified (β = –0.221, *P* < 0.05), suggesting that the relationship was moderated by psychological capital.

**TABLE 6:** Moderated regression analysis.

	Std. estimate	se	t	p	R2	F
Constant		6.275	13.917	0.000	0.238	52.271
Parental psychological control	–0.347	0.963	–11.974	0.000		
Psychological capital	–0.035	1.068	–1.228	0.220		
Parental psychological control x psychological capital	–0.221	1.277	–7.703	0.000		
Grade	–0.010	0.600	–0.379	0.705		
Gender	–0.091	1.729	–3.232	0.001		
Monthly income	0.035	0.658	1.230	0.219		

Dependent variable, physical activity.

**FIGURE 3:**
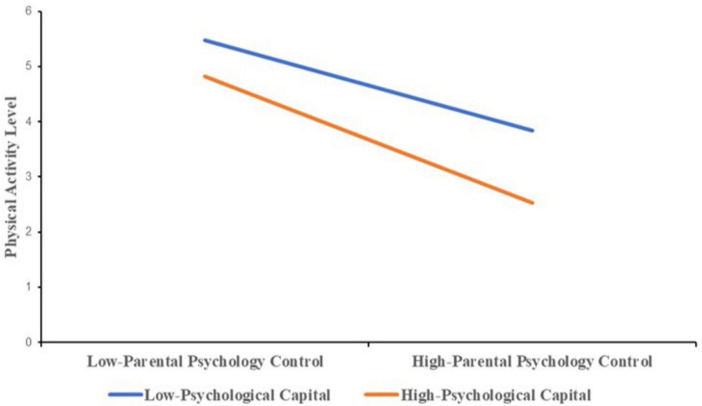
The cross-level moderation effect of psychological capital on the relationship between parental psychological control and physical activity level of adolescents.

The results in Table 7, obtained using the Bootstrap method with 5,000 resamples and a 95% confidence interval (CI), demonstrated significant moderating effects of psychological capital at various levels. At a low level (–1SD), the effect was –3.536 with a 95% CI of (–6.636, –0.436). At the mean level, the effect was –11.599 with a 95% CI of (–13.484, –9.714). At a high level (+ 1SD), the effect was –19.662 with a 95% CI of (–22.097, –17.226). None of the 95% confidence intervals included zero, indicating that as psychological capital increased, the negative impact of parental psychological control on adolescents’ physical activity was progressively attenuated, demonstrating a significant negative moderating effect (Figure 3).

**TABLE 7:** Bootstrap moderation effect test.

PC	Effect	se	*t*	*p*	95% CI	
					**Lower**	**Upper**
–1SD	–3.536	1.580	–2.238	0.025	–6.636	–0.436
Medium	–11.599	0.961	–12.076	0.000	–13.484	–9.714
+1SD	–19.662	1.241	–15.841	0.000	–22.097	–17.226

## 5 Discussion

The research outcomes highlighted a pronounced negative correlation between the psychological control exerted by parents and the physical activity levels of adolescents. Self-control was identified to partially mediate the relationship between parental psychological control and adolescent physical activity. Additionally, the involvement of psychological capital was found to exert a negative moderating effect on the adverse impact of parental psychological control on adolescent physical activity. These insights offered a novel lens through which to understand how psychological factors within the family setting influence adolescents. They facilitated an appreciation among parents of the pivotal role their behavior plays in shaping adolescent development, underscored the necessity of fostering self-control and psychological capital in children, and provided a scientific framework to guide family education.

### 5.1 The negative impact of parental psychological control on adolescent physical activity

Correlation analyses showed that there was a significant negative correlation between parental psychological control and adolescent physical activity (α = –0.311), indicating that higher levels of parental psychological control were associated with lower levels of adolescent physical activity participation, supporting Hypothesis 1 and consistent with former studies on the relationships between parental psychological control and health-related behaviors of adolescent (Lin et al., 2020; Tang et al., 2024). A prospective study demonstrated that the control exerted by parents through authoritative commands has a lasting negative impact on adolescents’ participation in moderate-to-vigorous physical activity (MVPA) (Doggui et al., 2021), supporting the conclusions of this study.

Attachment theory and self-determination theory provide theoretical frameworks to interpret these findings. According to attachment theory, parents are the primary objects of children’s early attachment relationships, providing a secure attachment base that enables children to feel secure while exploring their environment and engaging in activities. However, parents’ use of psychological control tactics, such as withdrawing love and inducing guilt, may undermine this secure attachment relationship. Such an insecure attachment relationship led adolescents to feel insecure and distrustful, resulting in negative psychological outcomes (Yaffe and Levental, 2024), which in turn affected their emotional regulation and behavioral choices. Self-Determination Theory posited that the three basic psychological needs of autonomy, competence, and relatedness are key factors in motivating individuals to self-activate and engage in a variety of activities (Flannery, 2017). Physical activities required autonomy support to enhance participants’ intrinsic motivation and willingness for sustained engagement (Fraguela-Vale et al., 2020). Parental psychological control may precipitate adverse psychological states that potentially undermine adolescents’ self-efficacy (Andretta and McKay, 2020), leading to diminished motivation for physical activity engagement (Blom et al., 2021), and consequently decreasing their likelihood of participation in such activities (Doré et al., 2020). As it turns out, the study also show that maladaptive educational approaches can have detrimental effects on adolescents’ physical activity participation.

While this study primarily highlights the significance of parental control within the microsystem, adolescents’ physical activity is influenced by interactions across broader environmental levels. From this perspective, Bronfenbrenner’s ecological systems theory (1979) offers a comprehensive framework for understanding these dynamics. For instance, psychological reflection within the microsystem may directly undermine adolescents’ engagement in physical activity, but mitigating factors in the mesosystem, such as positive peer relationships or school-based physical education policies, could help offset its negative effects (Moitra and Madan, 2022). Similarly, ecosystem-level factors, such as the availability of community-based sports programs, or macrosystem-level influences, like cultural norms promoting physical activity, further shape these outcomes (Duffey et al., 2021).

Future research should consider the dynamic interplay between these environmental systems to better understand how external factors interact with parental influences at different levels. For example, examining how socioeconomic conditions in the ecosystems or shifting societal norms in the macrosystem impact adolescents’ physical activity could provide more comprehensive insights into promoting health behaviors.

Compared to previous studies that primarily focused on the impact of psychological control on internalizing and externalizing problems in adolescents, less attention has been given to its effects on proactive health promotion behaviors. This study concentrates on the influence of detrimental parenting styles on physical activity, an essential health-promoting behavior. It not only broadens the scope of understanding the impact of parental psychological control but also provides new insights and empirical foundations for promoting healthy behaviors in adolescents.

### 5.2 The mediator role of self-control

The results of the mediation model in this study confirmed that self-control partially mediated the relationship between parental psychological control and adolescents’ physical activity. This indicates that parental psychological control not only directly affected the level of adolescents’ physical activity but also indirectly influenced it by affecting their self-control abilities. The study identified self-control as a key factor linking external control (parental psychological control) and personal behavior (adolescents’ physical activity), thereby supporting research hypothesis 2. Previous studies have shown that self-control mediates the relationship between parental psychological control and externalizing behaviors, highlighting the detrimental effect of parental psychological control on adolescents’ self-control. These findings align closely with the results of this study (Bai et al., 2020). Self-control has been shown to predict both actual exercise volume and exercise intentions (Gerdtham et al., 2020). Individuals with higher self-control abilities tend to engage in more physical activity (Boat et al., 2024), further supporting research hypothesis 2b. These findings underscore the significant role of self-control in promoting and maintaining physical activity behaviors among adolescents. Attachment theory and self-determination theory can also help explain these findings.

Parental psychological control disrupted the security of parent-child attachment by intruding upon and manipulating children’s psychological and emotional development. This led to feelings of helplessness and passivity in children (Inguglia et al., 2022), adversely affecting the development of their self-control abilities (Bai et al., 2020) and triggering maladaptive outcomes. According to self-determination theory, motivation was a key factor that drove individual behavior. On one hand, individuals with stronger self-control abilities perceived a clearer sense of autonomy (Mills and Allen, 2020). This autonomy support, in turn, contributed to increased engagement in physical activity (Fraguela-Vale et al., 2020). On the other hand, individuals with stronger self-control exhibited strong self-discipline and planning abilities, which were crucial in the past for maintaining their participation in physical activities, helping them overcome inertia and adhere to regular physical activity, thereby sustaining a healthy and sustainable lifestyle (Haible et al., 2020). Ultimately, the reduction of psychological control by parents, coupled with the enhancement of self-control skills in adolescents, is imperative for encouraging healthy behavioral selections among young people. Given the detrimental effects of parental psychological control on adolescents’ self-regulatory capacity, implementing differentiated interventions for educators and parents is imperative. Specifically, educators can facilitate weekly classroom-based workshops focusing on social-emotional competencies, including goal-setting strategies and stress management techniques. Concurrently, parents can foster autonomy through structured decision-making opportunities, such as allowing adolescents to select between athletic or artistic pursuits. These complementary intervention approaches may effectively enhance adolescents’ self-regulatory capabilities and promote their psychological wellbeing. This research extends the interconnectedness of parental psychological control, adolescents’ self-regulatory capacities, and health behaviors, presenting a fresh analytical framework for subsequent scholarly endeavors. It sheds light on the psychological processes through which family dynamics influence adolescents’ health, delivering substantial theoretical insights and practical applications for the realm of family education and the advancement of adolescent health initiatives.

### 5.3 Psychological capital as a moderator in alleviating the negative effects of parental psychological control on physical activity

Regression analysis results indicated that psychological capital exerted a negative moderating effect on the relationship between parental psychological control and adolescents’ physical activity, thereby confirming research hypothesis 3. Psychological capital, as a characteristic of a positive psychological state, was proven in this research to provide adolescents with an effective psychological buffer, enabling them to better withstand the negative effects of parental psychological control. Consistent with previous research findings on psychological capital as a positive coping mechanism, we highlighting its important role in mitigating negative effects (Maykrantz et al., 2021). Psychological capital encompassed psychological resources, including self-efficacy, hope, optimism, and resilience (Xiong et al., 2020), which enabled adolescents to maintain a positive sense of purpose and an optimistic perspective to cope with negative influences, even in situations where parents exerted psychological control (Bryce et al., 2020; Rincón Uribe et al., 2020). The role of psychological capital goes beyond merely boosting adolescents’ intrinsic motivation for physical activity; it also aids in better stress and emotion management, which is crucial in fostering increased participation in physical activities. Research indicated that optimistic and positive adolescents tended to engage in more sports and had relatively higher levels of physical activity (Andretta and McKay, 2020; Bottolfs et al., 2020; Özer et al., 2024). When parents endeavored to exert psychological control over their children’s behavior, adolescents possessing elevated levels of psychological capital demonstrated a superior capacity to navigate the stressors inherent in parent-child dynamics (Li et al., 2022). This resilience not only safeguarded their psychological wellbeing but also preserved their motivation and capability to engage in physical activities, even amidst challenging familial circumstances.

The core components of psychological capital—hope, self-efficacy, resilience, and optimism—can be significantly developed through systematic interventions. Studies reveal that strategies for enhancing psychological capital, while widely utilized in business settings, also provide notable advantages in education, sports, and psychotherapy (Yıldırım et al., 2025). Furthermore, research has confirmed that higher levels of psychological capital effectively buffer the detrimental effects of parental control on adolescents’ mental health, underscoring its protective role and critical importance (Loeb et al., 2021; Finch et al., 2023). When parents attempted to influence their children’s behavior through psychological control, adolescents with higher psychological capital were better equipped to cope with the stress within parent-child relationships. This not only protected their psychological wellbeing, but also maintained their motivation and ability to continue participating in physical activities even in adverse family environments. Moreover, elevated levels of self-efficacy and resilience imbue adolescents with a profound confidence in their capacity to engage in physical activities, thereby attenuating the adverse effects of external control on their behavioral choices (Di Maio et al., 2021; Shengyao et al., 2024). By sustaining this optimistic mindset, adolescents are empowered to exercise greater autonomy in selecting healthy lifestyles, even in the face of psychological manipulation from their parents.

To enhance adolescents’ psychological capital, policymakers could implement community support programs and school-based mental health education to establish a systematic external support network. For instance, schools could integrate resilience training or courses focused on developing psychological capital, equipping adolescents with stronger coping mechanisms to manage stress. Additionally, community-level initiatives, such as family education promotion programs, can help parents understand the potential harms of psychological control and encourage healthier parent-child interactions. These multi-level approaches collectively contribute to fostering the development of psychological capital in adolescents. This practical emphasis on nurturing psychological capital aligns with the theoretical framework established in this study, highlighting its vital role in mitigating the adverse effects of parental psychological control and promoting long-term healthful behaviors. This practical emphasis on nurturing psychological capital aligns with the theoretical framework established in this study, highlighting its vital role in mitigating the adverse effects of parental psychological control and promoting long-term healthful behaviors.

This study offers a novel theoretical framework for elucidating the intricate interplay between familial environments and individual psychological attributes, underscoring the critical importance of fostering and enhancing adolescents’ psychological capital within the familial context as a potentially efficacious strategy for promoting healthful behaviors. Based on the above, exploring the development of effective intervention programs targeting the risks of parental psychological control, such as enhancing psychological capital, and evaluating their effectiveness through empirical studies will be an important direction for future research. This will further address theoretical gaps in the field and provide practical guidance for promoting adolescent mental health and improving family education practices.

## 6 Conclusion

This investigation scrutinized the repercussions of parental psychological control on adolescent physical activity, underscoring the intermediary role of self-control and the modulatory impact of psychological capital. The findings revealed a markedly adverse correlation between parental psychological control and adolescent physical activity levels. Self-control was identified as a mitigating mediator in this nexus, and psychological capital was found to substantially ameliorate the negative repercussions of parental psychological control on physical activity. This study offers innovative perspectives on the complex interplay of psychological determinants influencing adolescent growth within familial milieus, accentuating the critical significance of parental conduct and establishing a solid theoretical framework for the development of family educational programs and prospective intervention strategies.

## 7 Limitation

Despite the commendable efforts undertaken in this research to investigate familial factors that may enhance physical activity levels among adolescents, several limitations persist. This study utilized self-reported questionnaires for data collection, which may introduce certain subjective biases, including social desirability bias and recall bias. Social desirability bias may lead participants to over report their physical activity levels to align with societal norms or expectations, while recall bias could result in underreporting or inaccurate reporting of physical activity due to difficulty remembering past behaviors. Although measures were implemented in the study design to mitigate the effects of these potential biases, self-reported data inherently have certain limitations. Future research could integrate objective measurement technologies, such as wearable devices, to complement self-reported data and achieve a more comprehensive and accurate assessment of physical activity behaviors. Moreover, the cross-sectional design of this investigation did not capture temporal variations; thus, subsequent research should consider longitudinal tracking to attain a more nuanced understanding of the enduring impacts of familial factors on adolescent physical activity.

Given the geographical and cultural limitations of the current sample, future research should broaden its cultural scope to more thoroughly elucidate the mechanisms underlying the relationship between familial influences and adolescent physical activity. Additionally, future studies should consider incorporating a wider range of family ecological factors to develop more comprehensive theoretical models and examine their applicability across diverse populations, thereby enriching the theoretical framework and expanding its practical implications. Consequently, future research must delve deeper into the impact of familial factors on adolescent physical activity within diverse cultural contexts, aiming to achieve a more holistic understanding of this complex and multifaceted issue.

## Data Availability

The original contributions presented in this study are included in this article/Supplementary material, further inquiries can be directed to the corresponding author.
