# Supplementary material for: The impact of parental psychological control on adolescents’ physical activity: the mediating role of self-control and the moderating role of psychological capital
Source: Front Psychol. 2025 May 14;16:1501720. doi: 10.3389/fpsyg.2025.1501720 (PMC12116588; doi:10.3389/fpsyg.2025.1501720)
Supplement: Supplementary file 1 [file Supplementary_file_1.docx]

# Supplementary Material

1、 Reliability analysis

Although the questionnaires used in this study have been thoroughly validated for reliability and validity, the study still conducted an analysis, and the results are as s-table 1, The reliability of the variables in this study ranges from 0.826 to 0.928, indicating that the research scales and variables have a high degree of reliability, demonstrating good stability and consistency.

S-Table 1 Reliability analysis results

|  | Alpha | Item number |
| --- | --- | --- |
| Parental psychological control | 0.946 | 18 |
| Self-control | 0.864 | 7 |
| Psychological capacity | 0.940 | 26 |

2、CFA

1）Parental psychological control questionnaire


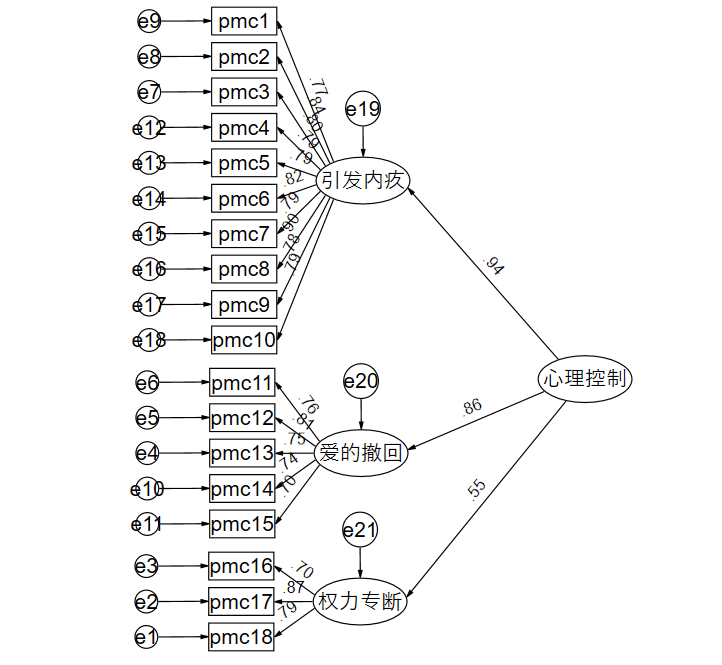


S-Table 2 Model Fit Results

|  | Reasonable Standard | Excellent Standard | Model Valu | Judgment | result |
| --- | --- | --- | --- | --- | --- |
| CMIN | 565.053 | | | | |
| CMIN/DF | <5 | <3 | 4.281 | Excellent | accept |
| GFI | >0.8 | >0.9 | 0.949 | Excellent | accept |
| AGFI | >0.8 | >0.9 | 0.933 | Excellent | accept |
| NFI | >0.8 | >0.9 | 0.956 | Excellent | accept |
| IFI | >0.8 | >0.9 | 0.966 | Excellent | accept |
| TLI | >0.8 | >0.9 | 0.960 | Excellent | accept |
| CFI | >0.8 | >0.9 | 0.966 | Excellent | accept |
| RMSEA | <0.08 | <0.05 | 0.057 | Excellent | accept |

It can be seen from the s-table 2: the model parameters all meet the reasonable standards, indicating that the model fit is up to the mark, and the model is acceptable.

S-Table 3 CR and AVE

|  |  |  | factor loading | R2 | AVE | CR |
| --- | --- | --- | --- | --- | --- | --- |
| Love withdrawal | <--- | Parental psychological control | 0.863 | 0.745 | 0.647 | 0.840 |
| Authority assertion | <--- | Parental psychological control | 0.552 | 0.305 |  |  |
| Guilt induction | <--- | Parental psychological control | 0.944 | 0.891 |  |  |
| pmc1 | <--- | Guilt induction | 0.773 | 0.598 | 0.652 | 0.949 |
| pmc2 | <--- | Guilt induction | 0.836 | 0.699 |  |  |
| pmc3 | <--- | Guilt induction | 0.803 | 0.645 |  |  |
| pmc4 | <--- | Guilt induction | 0.794 | 0.630 |  |  |
| pmc5 | <--- | Guilt induction | 0.785 | 0.616 |  |  |
| pmc6 | <--- | Guilt induction | 0.818 | 0.669 |  |  |
| pmc7 | <--- | Guilt induction | 0.791 | 0.626 |  |  |
| pmc8 | <--- | Guilt induction | 0.897 | 0.805 |  |  |
| pmc9 | <--- | Guilt induction | 0.780 | 0.608 |  |  |
| pmc10 | <--- | Guilt induction | 0.788 | 0.621 |  |  |
| pmc11 | <--- | Love withdrawal | 0.765 | 0.585 | 0.572 | 0.869 |
| pmc12 | <--- | Love withdrawal | 0.815 | 0.664 |  |  |
| pmc13 | <--- | Love withdrawal | 0.754 | 0.569 |  |  |
| pmc14 | <--- | Love withdrawal | 0.742 | 0.551 |  |  |
| pmc15 | <--- | Love withdrawal | 0.700 | 0.490 |  |  |
| pmc16 | <--- | Authority assertion | 0.696 | 0.484 | 0.623 | 0.831 |
| pmc17 | <--- | Authority assertion | 0.870 | 0.757 |  |  |
| pmc18 | <--- | Authority assertion | 0.792 | 0.627 |  |  |

From the table, it can be seen that the factor loadings of all variables range from 0.568 to 0.871; the composite reliability ranges from 0.831 to 0.921; and the average variance extracted ranges from 0.532 to 0.623. This indicates that all parameters meet the requirements for the structural model, suggesting that the internal quality of the model is acceptable

2）Self-control questionnaire


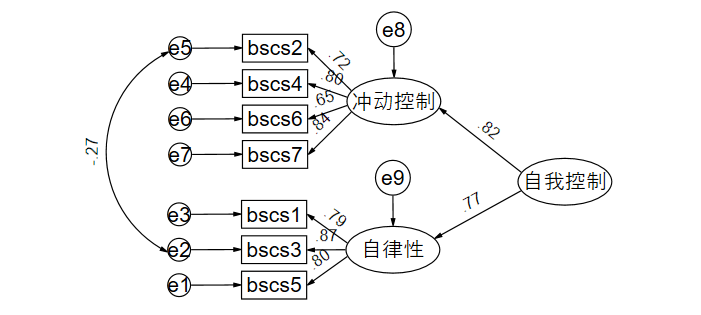


S-Table 4 Model Fit Results

|  | Reasonable Standard | Excellent Standard | Model Valu | Judgment | result |
| --- | --- | --- | --- | --- | --- |
| CMIN | 55.478 | | | | |
| CMIN/DF | <5 | <3 | 4.623 | Reasonable | accept |
| GFI | >0.8 | >0.9 | 0.985 | Excellent | accept |
| AGFI | >0.8 | >0.9 | 0.965 | Excellent | accept |
| NFI | >0.8 | >0.9 | 0.984 | Excellent | accept |
| IFI | >0.8 | >0.9 | 0.987 | Excellent | accept |
| TLI | >0.8 | >0.9 | 0.978 | Excellent | accept |
| CFI | >0.8 | >0.9 | 0.987 | Excellent | accept |
| RMSEA | <0.08 | <0.05 | 0.060 | Reasonable | accept |

It can be seen from the s-table 4: the model parameters all meet the reasonable standards, indicating that the model fit is up to the mark, and the model is acceptable.

S-Table 5 CR and AVE

|  |  |  | factor loading | R2 | AVE | CR |
| --- | --- | --- | --- | --- | --- | --- |
| Self-discipline | <--- | Self-control | 0.771 | 0.594 | 0.632 | 0.774 |
| Impulse control | <--- | Self-control | 0.817 | 0.667 |  |  |
| bscs5 | <--- | Self-discipline | 0.802 | 0.643 | 0.674 | 0.861 |
| bscs3 | <--- | Self-discipline | 0.868 | 0.753 |  |  |
| bscs1 | <--- | Self-discipline | 0.790 | 0.624 |  |  |
| bscs4 | <--- | Impulse control | 0.801 | 0.642 | 0.572 | 0.841 |
| bscs2 | <--- | Impulse control | 0.716 | 0.513 |  |  |
| bscs6 | <--- | Impulse control | 0.653 | 0.426 |  |  |
| bscs7 | <--- | Impulse control | 0.840 | 0.706 |  |  |

From the table, it can be seen that the factor loadings of all variables range from 0.606 to 0.932; the composite reliability ranges from 0.791 to 0.815; and the average variance extracted ranges from 0.532 to 0.560. This indicates that all parameters meet the requirements for the structural model, suggesting that the internal quality of the model is acceptable.

3）Psychological capacity questionnaire


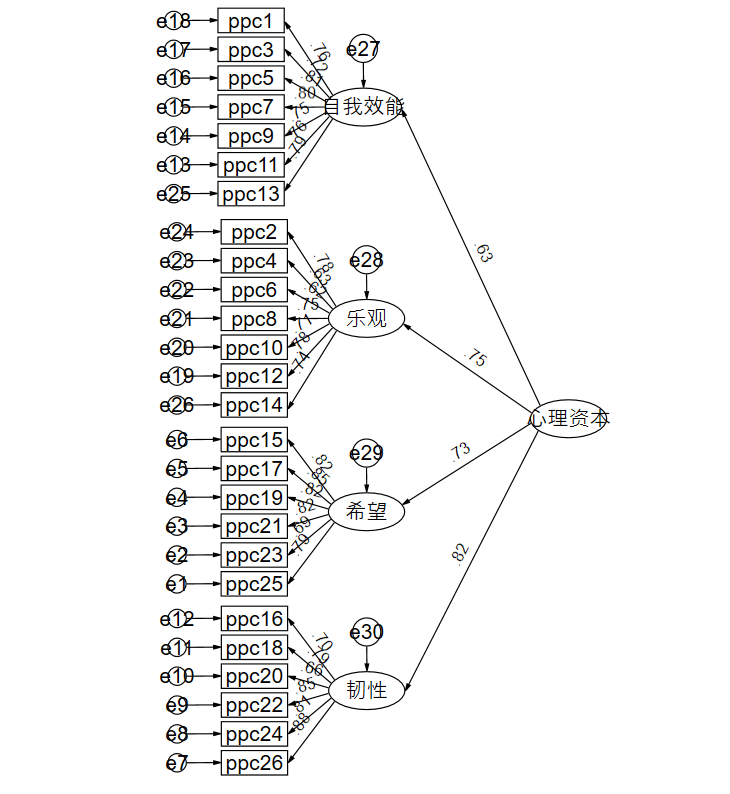


S-Table 6 Model Fit Results

|  | Reasonable Standard | Excellent Standard | Model Valu | Judgment | result |
| --- | --- | --- | --- | --- | --- |
| CMIN | 722.741 | | | | |
| CMIN/DF | <5 | <3 | 2..450 | Excellent | accept |
| GFI | >0.8 | >0.9 | 0.953 | Excellent | accept |
| AGFI | >0.8 | >0.9 | 0.944 | Excellent | accept |
| NFI | >0.8 | >0.9 | 0.955 | Excellent | accept |
| IFI | >0.8 | >0.9 | 0.973 | Excellent | accept |
| TLI | >0.8 | >0.9 | 0.970 | Excellent | accept |
| CFI | >0.8 | >0.9 | 0.973 | Excellent | accept |
| RMSEA | <0.08 | <0.05 | 0.038 | Excellent | accept |

It can be seen from the s-table 6: the model parameters all meet the reasonable standards, indicating that the model fit is up to the mark, and the model is acceptable.

S-Table 6: CR and AVE

|  |  |  | factor loading | R2 | AVE | CR |
| --- | --- | --- | --- | --- | --- | --- |
| Optimism | <--- | Psychological capacity | 0.754 | 0.569 | 0.545 | 0.826 |
| Hope | <--- | Psychological capacity | 0.730 | 0.533 |  |  |
| Resilience | <--- | Psychological capacity | 0.823 | 0.677 |  |  |
| Self-efficacy | <--- | Psychological capacity | 0.634 | 0.402 |  |  |
| ppc23 | <--- | Hope | 0.686 | 0.471 | 0.638 | 0.913 |
| ppc21 | <--- | Hope | 0.818 | 0.669 |  |  |
| ppc19 | <--- | Hope | 0.823 | 0.677 |  |  |
| ppc17 | <--- | Hope | 0.846 | 0.716 |  |  |
| ppc25 | <--- | Hope | 0.791 | 0.626 |  |  |
| ppc15 | <--- | Hope | 0.820 | 0.672 |  |  |
| ppc24 | <--- | Resilience | 0.809 | 0.654 | 0.819 | 0.906 |
| ppc22 | <--- | Resilience | 0.853 | 0.728 |  |  |
| ppc20 | <--- | Resilience | 0.660 | 0.436 |  |  |
| ppc18 | <--- | Resilience | 0.786 | 0.618 |  |  |
| ppc26 | <--- | Resilience | 0.883 | 0.780 |  |  |
| ppc16 | <--- | Resilience | 0.704 | 0.496 |  |  |
| ppc11 | <--- | Self-efficacy | 0.758 | 0.575 | 0.594 | 0.911 |
| ppc13 | <--- | Self-efficacy | 0.792 | 0.627 |  |  |
| ppc1 | <--- | Self-efficacy | 0.764 | 0.584 |  |  |
| ppc9 | <--- | Self-efficacy | 0.753 | 0.567 |  |  |
| ppc7 | <--- | Self-efficacy | 0.797 | 0.635 |  |  |
| ppc5 | <--- | Self-efficacy | 0.807 | 0.651 |  |  |
| ppc3 | <--- | Self-efficacy | 0.723 | 0.523 |  |  |
| ppc12 | <--- | Optimism | 0.780 | 0.608 | 0.516 | 0.881 |
| ppc10 | <--- | Optimism | 0.707 | 0.500 |  |  |
| ppc8 | <--- | Optimism | 0.754 | 0.569 |  |  |
| ppc6 | <--- | Optimism | 0.616 | 0.379 |  |  |
| ppc4 | <--- | Optimism | 0.626 | 0.392 |  |  |
| ppc2 | <--- | Optimism | 0.783 | 0.613 |  |  |
| ppc14 | <--- | Optimism | 0.743 | 0.552 |  |  |

From the table, it can be seen that the factor loadings of all variables range from 0.619 to 0.883; the composite reliability ranges from 0.881 to 0.914; and the average variance extracted ranges from 0.516 to 0.639. This indicates that all parameters meet the requirements for the structural model, suggesting that the internal quality of the model is acceptable.

4 Discriminant validity

S-Table 7: discriminant validity

|  | 1 | 2 | 3 | 4 |
| --- | --- | --- | --- | --- |
| Parental psychological control | **0.804** |  |  |  |
| Self-control | -.302^**^ | **0.795** |  |  |
| Psychological capacity | -0.006 | 0.026 | **0.738** |  |
| Physical Activity | -.423^**^ | .358^**^ | -0.041 | - |

Note: Bold indicates the arithmetic root mean square of AVE; "-" indicates absence.

From the s-table 7, it can be observed that the arithmetic root mean square of AVE for all variables is greater than the correlations between adjacent row and column variables, indicating that the model has good discriminant validity.

5 Descriptive analysis supplement.

| S-Table 8 Grade | | | | | |
| --- | --- | --- | --- | --- | --- |
|  | | Frequence | % | Available% | Cumulative% |
| Valid | 7th Grade | 216 | 21.4 | 21.4 | 21.4 |
|  | 8th Grade | 202 | 20.0 | 20.0 | 41.4 |
|  | 9th Grade | 195 | 19.3 | 19.3 | 60.7 |
|  | 10th Grade | 209 | 20.7 | 20.7 | 81.4 |
|  | 11th Grade | 188 | 18.6 | 18.6 | 100.0 |
|  | Total | 1010 | 100.0 | 100.0 |  |

| S-Table 9 Gender | | | | | |
| --- | --- | --- | --- | --- | --- |
|  | | Frequence | % | Available% | Cumulative% |
| valid | Male | 492 | 48.7 | 48.7 | 48.7 |
|  | Female | 518 | 51.3 | 51.3 | 100.0 |
|  | total | 1010 | 100.0 | 100.0 |  |

| S-Table 10 Father's occupation | | | | | |
| --- | --- | --- | --- | --- | --- |
|  | | Frequence | % | Available% | Cumulative% |
| valid | Worker | 54 | 5.3 | 5.3 | 5.3 |
|  | Service personnel | 42 | 4.2 | 4.2 | 9.5 |
|  | Scientific, educational, cultural, health, and professional technical personnel | 88 | 8.7 | 8.7 | 18.2 |
|  | Staff of enterprises and institutions | 348 | 34.5 | 34.5 | 52.7 |
|  | Government officials | 87 | 8.6 | 8.6 | 61.3 |
|  | Individual entrepreneurs | 223 | 22.1 | 22.1 | 83.4 |
|  | Others | 168 | 16.6 | 16.6 | 100.0 |
|  | total | 1010 | 100.0 | 100.0 |  |

From the s-table 10, it can be seen that a total of 1,010 valid data entries were collected for this study. Among them, 348 individuals (34.5%) reported that their father's occupation was staff of enterprises and institutions, which is the most common. The second largest group consisted of 223 individuals (22.1%) whose fathers were individual entrepreneurs. Additionally, 168 individuals (16.6%) selected "others" for options not listed above. There were 88 individuals (8.7%) whose fathers were scientific, educational, cultural, health, and professional technical personnel; 87 individuals (8.6%) whose fathers were government officials; 54 individuals (5.3%) whose fathers were workers; and 42 individuals (4.2%) whose fathers were service personnel, which was the smallest group.

| S-Table 11 Mother's occupation | | | | | |
| --- | --- | --- | --- | --- | --- |
|  | | Frequence | % | Available% | Cumulative% |
| valid | Worker | 30 | 3.0 | 3.0 | 3.0 |
|  | Service personnel | 48 | 4.8 | 4.8 | 7.7 |
|  | Scientific, educational, cultural, health, and professional technical personnel | 129 | 12.8 | 12.8 | 20.5 |
|  | Staff of enterprises and institutions | 269 | 26.6 | 26.6 | 47.1 |
|  | Government officials | 72 | 7.1 | 7.1 | 54.3 |
|  | Individual entrepreneurs | 174 | 17.2 | 17.2 | 71.5 |
|  | Others | 288 | 28.5 | 28.5 | 100.0 |
|  | total | 1010 | 100.0 | 100.0 |  |

From the s-table11, it can be seen that a total of 1,010 valid data entries were collected for this study. Among them, 288 individuals (28.5%) reported that their mother's occupation was "others" for options not listed above, which is the most common response. The second largest group consisted of 269 individuals (26.6%) whose mothers were staff of enterprises and institutions. Additionally, there were 174 individuals (17.2%) whose mothers were individual entrepreneurs; 129 individuals (12.8%) whose mothers were scientific, educational, cultural, health, and professional technical personnel; 72 individuals (7.1%) whose mothers were government officials; 48 individuals (4.8%) whose mothers were service personnel; and 30 individuals (3%) whose mothers were workers, which was the smallest group.

| S-Table 12 Father's education level | | | | | |
| --- | --- | --- | --- | --- | --- |
|  | | Frequence | % | Available% | Cumulative% |
| valid | Junior high school and below | 115 | 11.4 | 11.4 | 11.4 |
|  | Vocational school or high school | 213 | 21.1 | 21.1 | 32.5 |
|  | Associate degree | 162 | 16.0 | 16.0 | 48.5 |
|  | Bachelor's degree | 422 | 41.8 | 41.8 | 90.3 |
|  | Master's degree | 69 | 6.8 | 6.8 | 97.1 |
|  | Doctorate | 29 | 2.9 | 2.9 | 100.0 |
|  | Total | 1010 | 100.0 | 100.0 |  |

It can be seen from the s-table 12 that a total of 1,010 valid data entries were collected in this study. There were 422 fathers with a bachelor's degree, accounting for 41.8%, which is the highest number; followed by 213 with vocational or high school education, accounting for 21.1%; 162 with an associate degree, accounting for 16%; 115 with junior high school or below, accounting for 11.4%; 69 with a master's degree, accounting for 6.8%; and 29 with a doctoral degree, accounting for 2.9%, which is the smallest number.

| S-Table 13 Father's education level | | | | | |
| --- | --- | --- | --- | --- | --- |
|  | | Frequence | % | Available% | Cumulative% |
| valid | Junior high school and below | 123 | 12.2 | 12.2 | 12.2 |
|  | Vocational school or high school | 182 | 18.0 | 18.0 | 30.2 |
|  | Associate degree | 177 | 17.5 | 17.5 | 47.7 |
|  | Bachelor's degree | 453 | 44.9 | 44.9 | 92.6 |
|  | Master's degree | 50 | 5.0 | 5.0 | 97.5 |
|  | Doctorate | 25 | 2.5 | 2.5 | 100.0 |
|  | Total | 1010 | 100.0 | 100.0 |  |

From the s-table 13, it can be seen that a total of 1,010 valid data entries were collected for this study. Among them, 453 individuals (44.9%) reported that their mother's education level was a bachelor's degree, which is the most common response. The second largest group consisted of 182 individuals (18%) whose mothers had a vocational school or high school education. Additionally, there were 177 individuals (17.5%) with an associate degree; 123 individuals (12.2%) with a junior high school education or below; 50 individuals (5%) with a master's degree; and 25 individuals (2.5%) with a doctorate, which was the smallest group.

| S-Table 14 Monthly Income | | | | | |
| --- | --- | --- | --- | --- | --- |
|  | | Frequence | % | Available% | Cumulative% |
| valid | Below 3000 | 37 | 3.7 | 3.7 | 3.7 |
|  | 3000-6000 | 110 | 10.9 | 10.9 | 14.6 |
|  | 6000-12000 | 323 | 32.0 | 32.0 | 46.5 |
|  | 12000-18000 | 238 | 23.6 | 23.6 | 70.1 |
|  | 18000-24000 | 154 | 15.2 | 15.2 | 85.3 |
|  | Above 24000 | 148 | 14.7 | 14.7 | 100.0 |
|  | total | 1010 | 100.0 | 100.0 |  |

| S-Table 15 physical activity item | | | | | |
| --- | --- | --- | --- | --- | --- |
|  | | Frequence | % | Available% | Cumulative% |
| valid | Walking, Running | 178 | 17.6 | 17.6 | 17.6 |
|  | Traveling, Hiking | 172 | 17.0 | 17.0 | 34.7 |
|  | Sports Dance | 143 | 14.2 | 14.2 | 48.8 |
|  | Ball Games | 398 | 39.4 | 39.4 | 88.2 |
|  | Skipping Rope | 19 | 1.9 | 1.9 | 90.1 |
|  | Tai Chi, Health Preservation Techniques | 9 | .9 | .9 | 91.0 |
|  | Fitness Equipment Activities | 15 | 1.5 | 1.5 | 92.5 |
|  | Swimming | 38 | 3.8 | 3.8 | 96.2 |
|  | Other | 38 | 3.8 | 3.8 | 100.0 |
|  | Total | 1010 | 100.0 | 100.0 |  |

It can be seen from the s-table15 that a total of 1,010 valid data entries were collected in this study. Among them, 398 people, accounting for 39.4%, prefer ball games for physical exercise, which is the highest number; followed by 178 people, accounting for 17.6%, who prefer walking and running; 172 people, accounting for 17%, enjoy traveling and hiking; 143 people, accounting for 14.2%, like sports dance; 38 people, accounting for 3.8%, prefer swimming; another 38 people, also accounting for 3.8%, choose other activities; 19 people, accounting for 1.9%, like skipping rope; 15 people, accounting for 1.5%, enjoy fitness equipment activities; and 9 people, accounting for 0.9%, prefer Tai Chi and health preservation techniques, which is the smallest number.

| S-Table 16 physical activity intensity | | | | | |
| --- | --- | --- | --- | --- | --- |
|  | | Frequence | % | Available% | Cumulative% |
|  | Light exercise | 177 | 17.5 | 17.5 | 17.5 |
|  | Low-intensity, not very strenuous exercise | 157 | 15.5 | 15.5 | 33.1 |
|  | Moderate-intensity, more intense and sustained exercise | 239 | 23.7 | 23.7 | 56.7 |
|  | High-intensity, short-duration exercise with rapid breathing and heavy sweating | 356 | 35.2 | 35.2 | 92.0 |
|  | High-intensity, sustained exercise with rapid breathing and heavy sweating | 81 | 8.0 | 8.0 | 100.0 |
|  | Total | 1010 | 100.0 | 100.0 |  |

| S-Table 17 physical activity time | | | | | |
| --- | --- | --- | --- | --- | --- |
|  | | Frequence | % | Available% | Cumulative% |
| valid | Less than 10 minutes | 59 | 5.8 | 5.8 | 5.8 |
|  | 11 to 20 minutes | 162 | 16.0 | 16.0 | 21.9 |
|  | 21 to 30 minutes | 283 | 28.0 | 28.0 | 49.9 |
|  | 31 to 59 minutes | 253 | 25.0 | 25.0 | 75.0 |
|  | More than 60 minutes | 253 | 25.0 | 25.0 | 100.0 |
|  | Total | 1010 | 100.0 | 100.0 |  |

| S-Table 17 physical activity frequence | | | | | |
| --- | --- | --- | --- | --- | --- |
|  | | Frequence | % | Available% | Cumulative% |
| valid | Less than once a month | 43 | 4.3 | 4.3 | 4.3 |
|  | 3 to 5 times a week | 312 | 30.9 | 30.9 | 35.1 |
|  | 2 to 3 times a month | 150 | 14.9 | 14.9 | 50.0 |
|  | About once a day | 166 | 16.4 | 16.4 | 66.4 |
|  | 1 to 2 times a week | 339 | 33.6 | 33.6 | 100.0 |
|  | Total | 1010 | 100.0 | 100.0 |  |
